# Concept description and accuracy evaluation of a moldable surgical targeting system

**DOI:** 10.1117/1.JMI.8.1.015003

**Published:** 2021-02-19

**Authors:** Thomas S. Rau, Sina Witte, Lea Uhlenbusch, Lüder A. Kahrs, Thomas Lenarz, Omid Majdani

**Affiliations:** a Hannover Medical School, Department of Otolaryngology, Cluster of Excellence EXC 2177/1 “Hearing4all”, Hannover, Germany; b University of Toronto Mississauga, Department of Mathematical and Computational Sciences, Mississauga, Ontario, Canada; c Hospital for Sick Children (SickKids), Centre for Image Guided Innovation and Therapeutic Intervention, Toronto, Ontario, Canada

**Keywords:** cochlear implantation, image-guided surgery, micro-stereotactic frame, minimally invasive surgery, surgical template

## Abstract

**Purpose:** We explain our concept for customization of a guidance instrument, present a prototype, and describe a set of experiments to evaluate its positioning and drilling accuracy.

**Methods:** Our concept is characterized by the use of bone cement, which enables fixation of a specific configuration for each individual surgical template. This well-established medical product was selected to ensure future intraoperative fabrication of the template under sterile conditions. For customization, a manually operated alignment device is proposed that temporary defines the planned trajectory until the bone cement is hardened. Experiments (n=10) with half-skull phantoms were performed. Analysis of accuracy comprises targeting validations and experiments including drilling in bone substitutes.

**Results:** The resulting mean positioning error was found to be 0.41±0.30  mm at the level of the target point whereas drilling was possible with a mean accuracy of 0.35±0.30  mm.

**Conclusion:** We proposed a cost-effective, easy-to-use approach for accurate instrument guidance that enables template fabrication under sterile conditions. The utilization of bone cement was proven to fulfill the demands of an easy, quick, and prospectively intraoperatively doable customization. We could demonstrate sufficient accuracy for many surgical applications, e.g., in neurosurgery. The system in this early development stage already outperforms conventional stereotactic frames and image-guided surgery systems in terms of targeting accuracy.

## 1 Introduction

Accurate positioning and orientation of surgical tools, such as stimulation probes, drills, or biopsy needles, require assistance systems that support the surgeon in performing these demanding tasks. This need of assistance applies in particular if the anatomical conditions necessitate an accuracy of a few tenths of a millimeter and if the target region is located invisibly deep inside the body. One prominent example for high accuracy requirements is cochlear implant surgery (CIS) performed in a minimally invasive manner, i.e., replacing the conventional approach by a single drill hole to get access to the inner ear (cochlea).

Due to the major technical challenges, minimally invasive CI surgery (minCIS) has been under development for more than one decade since the pioneer study by Labadie et al.^1^ published in 2005. As the cochlea is embedded in non-elastic tissue (bone) deep inside the lateral skull base, and a very small target region of <1.6  mm in diameter^2^ must be reached, only tiny inaccuracies are allowed. It is impossible to perform the drilling procedure completely manually. On the contrary, highly accurate positioning of the drilling tool along a previously planned trajectory is obligatory based on patient-specific imaging.

Within the last years, different concepts of surgical assistance systems have been developed and tested up to different levels of maturity ranging from only conceptual designs, to preclinical evaluation to even first clinical studies.^3^^–^^5^ From a technical point of view these concepts include

• stationary robot systems;^3^^,^^6^^–^^8^
• head mounted robot systems;^9^^,^^10^
• micro-stereotactic frames (MSFs).^4^^,^^11^^–^^13^

Common features of the last-mentioned MSFs are (1) bone-anchored fixation at the patient’s skull roughly near the desired drill path, (2) individual fabrication or adjustment of at least one component, and (3) their high stiffness to serve as rigid drill guides. Several different concepts of MSFs have been described in literature in which customization is implemented, e.g., by means of 3D printing,^5^^,^^11^ by milling using computer numerical control (CNC) machining,^14^^,^^15^ or by length and shape setting of at least three legs^12^^,^^13^ to align the main axis of the instrument guide with the patient-specific planned drilling trajectory.

Although there are already some good approaches, we believe that fabrication of individual instrument guides can be made even easier and faster. Of particular importance, from our point of view, is that the customization can be done directly in the operating room (OR) by the surgical team under sterile conditions to omit the time-consuming sterilization procedure prior to or during surgery. This requires an easy-to-use method for intraoperative finalization. Bone cement is a material that is well-known to medical professionals, easy to use, available as a sterile medical product (which later simplifies conformity with the regulatory requirements), is moldable to enable adaption to the individually planned trajectory, and hardens within a few minutes to lock the patient-specific configuration. In addition to the above-mentioned experimental concepts, in dental implantation, so-called surgical templates are well established.^16^^,^^17^ They are individually fabricated and serve as instrument guides for drillings into the mandible or maxillary while using, e.g., the surrounding teeth as rigid and one-to-one fixation points. As comparable protruding natural landmarks are not available at the lateral surface of the temporal bone, at least one artificial supporting structure is required serving both as reference object and as mounting base for the customized surgical template. This part is referred to as “reference frame” in the following, whereas “surgical template,” and “instrument guide” are used synonymously for the individualized component of the MSF. Both surgical template and reference frame together form the MSF, herein also referred to as “surgical targeting system.”

This journal paper concludes and extends our previous conference papers^18^^,^^19^ and describes in detail the implementation of the concept. For the first time, we describe to the full extent our methodology with verification experiments and results. In contrast to the earlier conference papers, the new system is now evaluated using half-skull phantoms. Thereby, not only positioning but also drilling accuracy was determined while parts of the surgical workflow were imitated by resembling the clinical environment.^20^ Furthermore, we advanced our measurement methods of the positioning accuracy to achieve more accurate and reliable results. In the following, we provide detailed insights into design specifications of the whole system, its current implementation, and discuss our considerations and decisions within the design and development process with respect to previous findings from literature.

## 2 Materials and Methods

### 2.1 Description of the System

#### 2.1.1 Reference frame

**Fig. 1:**
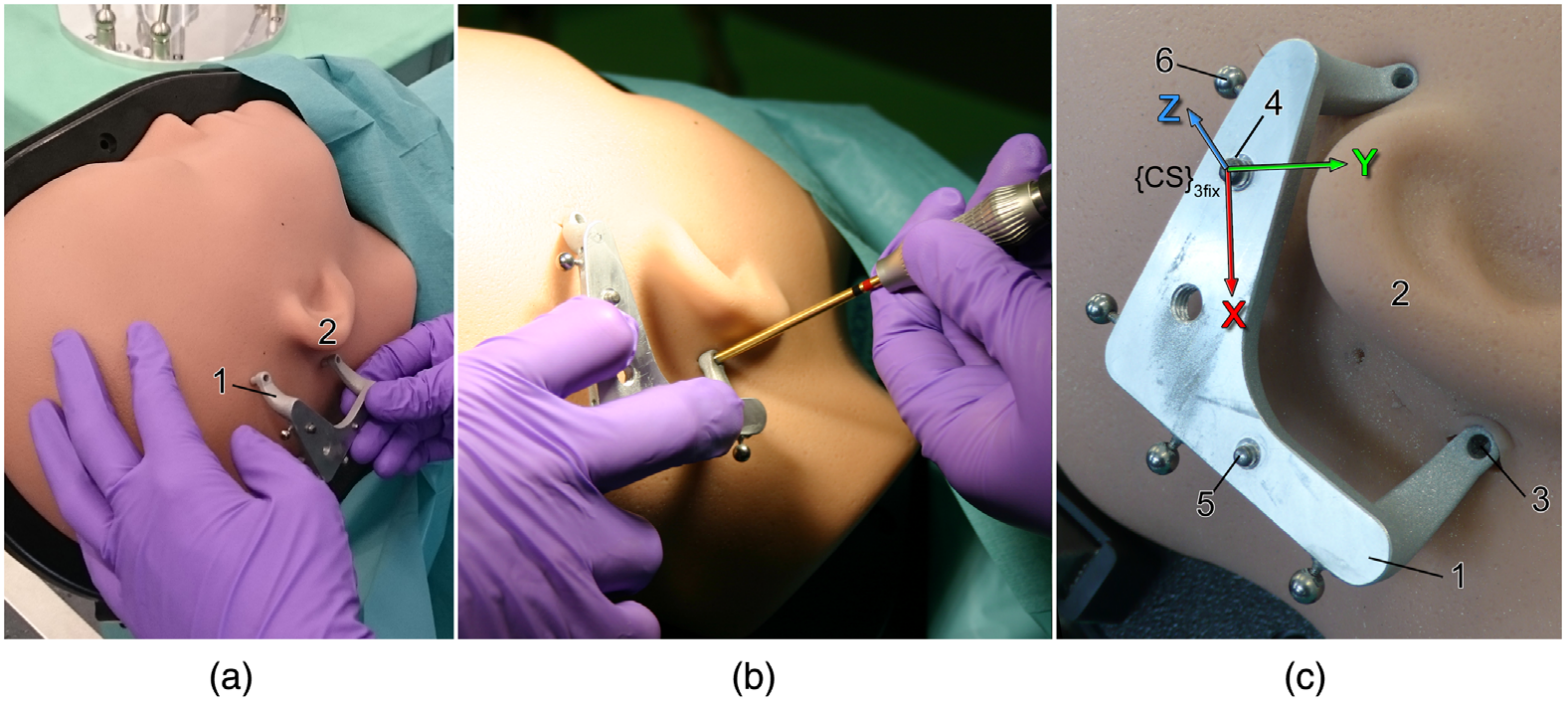
(a) The reference frame (1, Trifix) is positioned behind the pinna (2) considering the expected drill path. (b) It is bone-anchored through small skin incisions using three bone screws (3). (c) The MCI, consisting of the planar top face of the frame and two dowel pins (4, 5), enables mounting of the surgical template. It is also used to the define the MCS ${\left\{\mathrm{CS}\right\}}_{3\text{fix}}$ with the origin in the center of the larger dowel pin, the planar top face being the xy-plane, and the x axis being oriented toward the smaller dowel pin. For image-to-patient registration, the Trifix is equipped with four titanium spheres (6).

The envisaged surgical workflow requires a reference frame that has to be rigidly fixed on the patient’s skull (Fig. 1). This bone-anchored part of the system serves as temporary but rigid connector between the anatomical target structure and the instrument guide. This mechanical fixation to the bone is realized by use of three small, self-tapping bone screws (Max Drive Drill Free 2×9, KLS Martin Group, Tuttlingen, Germany) through small skin incisions. Screw-in depth is limited to 3.6 mm. Due to the number of bone anchors this type of reference frame is referred to as “Trifix” and “3fix” in the following.

On the upper surface of the Trifix, the rigid fixation of the surgical template is ensured by a definite mechanical coupling interface (MCI). In the current prototype, the MCI is defined by the planar top face of the reference frame and two dowel pins for accurate mounting of the counterparts without clearance. A screw secures the connection.

The reference frame is used to define a master coordinate system (MCS) ${\left\{\mathrm{CS}\right\}}_{3\text{fix}}$; both in physical (Fig. 1) and image space. For an improved registration procedure, the Trifix was equipped with four titanium spheres (Ø5  mm). The positions of these spherical registration markers were measured in ${\left\{\mathrm{CS}\right\}}_{3\text{fix}}$ using a portable coordinate measurement machine (CMM, Romer Absolute Arm Compact 7312, Hexagon Manufacturing Intelligence, Wetzlar, Germany with a volumetric accuracy of 0.025 mm). As the reference frame and especially its spherical fiducials are clearly visible in CT images, the MCS can be identified in the image space, enabling image-to-patient registration.

#### 2.1.2 Surgical template

**Fig. 2:**
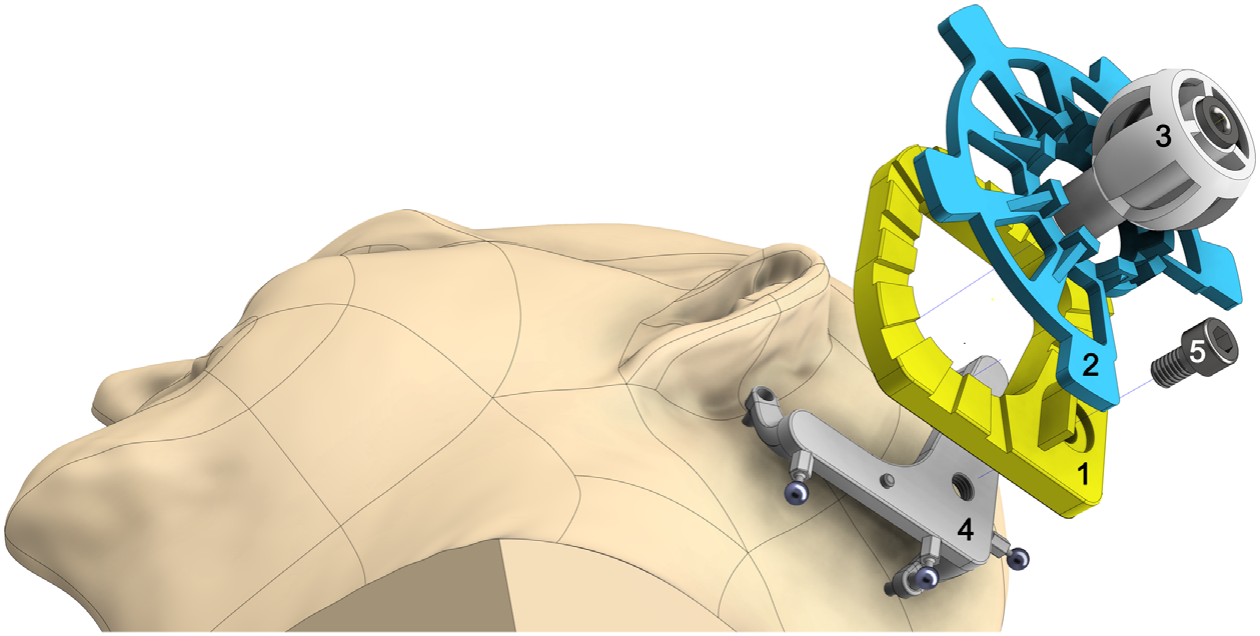
Schematic drawing of the moldable surgical targeting system. Base plate (1), subcarrier (2), and drill bushing holder (3) forms the individual component while the bone-attached reference frame (4, Trifix) is the reusable part. Dowel pins and a screw (5) secure the connection.

As the reference frame is designed to suit most patients, our surgical targeting system requires an individual component, which strictly constrains the surgical tool movements along the patient-specific planned trajectory. A unique feature of the present concept is that the surgical template is glued together in the desired, patient-specific configuration using few separate parts and bone cement. In its current design, it will be composed of a “base plate,” an intermediate element referred to as “subcarrier,” and a “drill bushing holder” (Fig. 2).

The interface between the drill bushing holder and the subcarrier serves as a ball-and-socket joint having three degrees of freedom (DOF); the subcarrier and the base plate form a planar joint with three DOF. As the axis of rotation between the subcarrier and the base plate is redundant to one rotational DOF of the ball joint, the total system covers five DOF. Only the translation in the direction of the trajectory cannot be controlled by the surgical template in its current design. Therefore, an additional depth control is required to position the guided instrument at a specific depth.

The underside of the base plate is designed as counterpart to the MCI including corresponding holes for the dowel pins. The upper side is structured by several grooves to provide a larger and more complex surface. The idea behind that design is to enable the bone cement to infiltrate all cavities and to interlock the separate parts for optimal stability and permanent fixation.

For the current prototypes headed drill bushings (steel, hardened, according to DIN 172, type 4×7×16) have been used as linear guide. Two of them are stuck into the drill bushing holder. That holder shows a skeleton-like design with resulting hollows for infiltration of the bone cement for a form-locking connection.

#### 2.1.3 Jig Maker

**Fig. 3:**
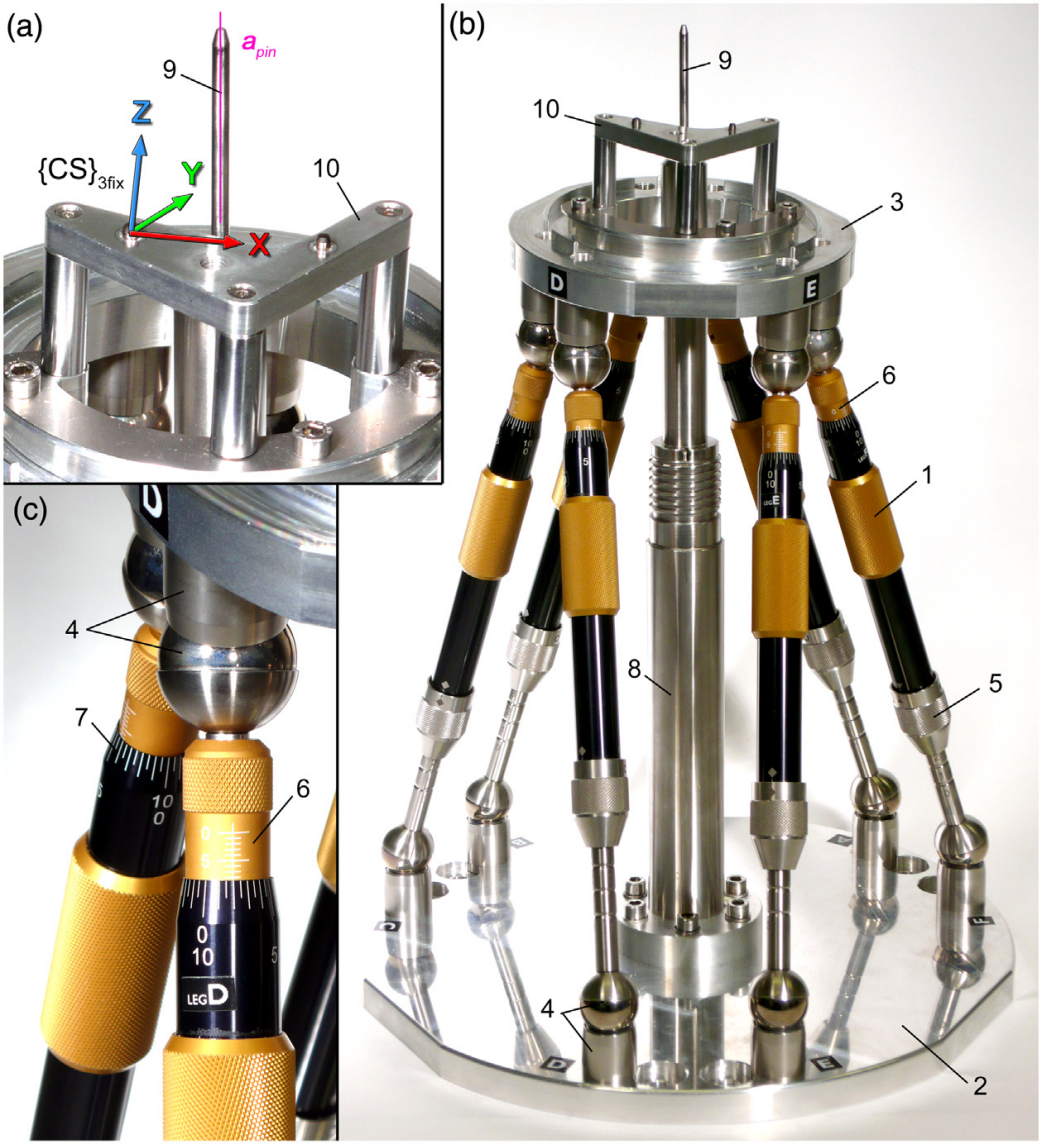
The Jig Maker. The pose setting device consists of six prismatic joints (1) which connect a ground plate (2) with a moving platform (3) using magnetic ball joints (4). Each strut has an interlock mechanism for rough length setting (5) and a micrometer screw (6) with Vernier scale (7) for fine adjustment. A central pillar (8) ends in an alignment pin (9) representing the planned trajectory $a_{\text{pin}}$. A mounting table (10) on top of the moving platform features the same MCI and therefore referred to as ${\left\{\mathrm{CS}\right\}}_{3\text{fix}}$ as well.

Individualization of the surgical template requires a device that temporarily constrains the drill bushings along the planned trajectory until the aligned parts are permanently glued by the bone cement. The design of the alignment device, hereafter referred to as “Jig Maker” (Fig. 3), was adapted from the guiding sleeve positioning hexapod system (X1med3D, Schick GmbH, Schemmerhofen, Germany), which can be purchased for dental purposes.^16^^,^^17^ In contrast to a previous version,^18^ only the passive prismatic joints of the hexapod were utilized whereas the complete mechanism was newly re-designed (using Inventor, Professional 2017, Autodesk, San Rafael, California). The Jig Maker is designed as a Gough-Stewart platform including six passive prismatic joints (in the following referred to as “struts”), which connect a ground plate with a moving platform, the top plate. The movable connection to both plates is realized by steel spheres (Ø25  mm) at both ends of the struts interacting in magnetic joint sockets. The length of each strut can be manually adjusted. A mechanism enables rough setting of the length in ten steps of 10 mm; in addition, each leg features a micrometer screw for fine adjustment (with increments of 0.05 mm on the rotating Vernier scale). The pose (position and orientation) of the moving top plate can be adjusted by changing the length $\lambda_{i}$ of the struts (with i∈{A,B,…,F}). Similar indices were used for their corresponding magnetic sockets at both ground $G_{i,0}$ and top $G_{i,1}$ plate.

A pillar is mounted in the center of the ground plate and its main axis $a_{\text{pin}}$ corresponds to the planned trajectory $t_{\text{plan}}$ (bold italic serif letters represent vectors). Due to stiffness reasons, the diameter of the pillar decreases from base to the top, ending in an alignment pin with 4 mm in diameter, which fits to the drill bushings of the surgical template. The top plate has a mounting table that features the identical MCI as the Trifix. Consequently, the base plate of the surgical template can be mounted at the same position with respect to the MCS.

**Fig. 4:**
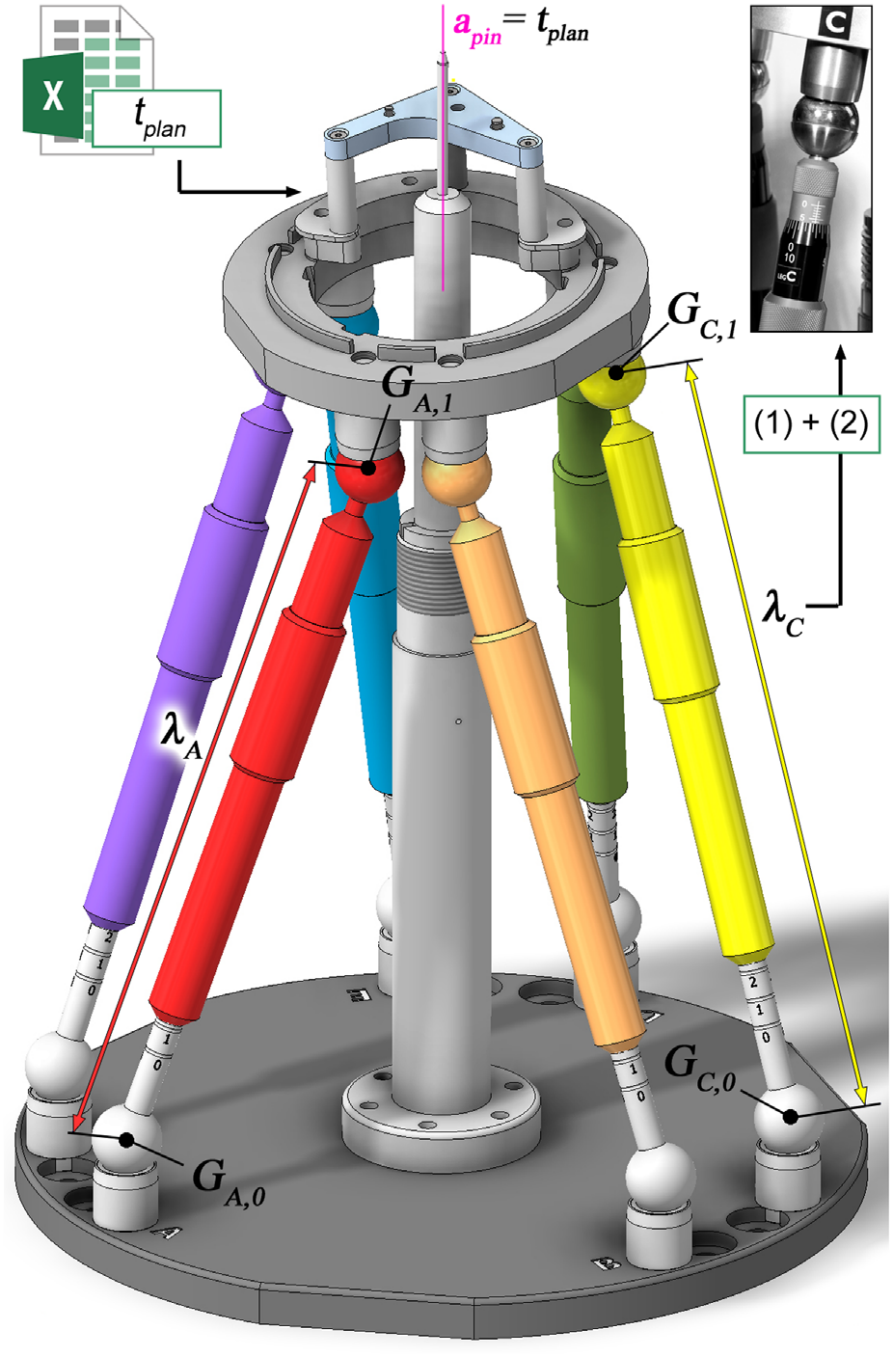
A virtual CAD assembly model of the pose setting device was used to determine the strut lengths based on the planned trajectory. Coordinates of the trajectory ${\overset{\to }{t}}_{\text{plan}}$ are stored in an Excel spreadsheet that drives the parametric Jig Maker CAD model. Driven dimensions between the centers of the ball joints provide lengths of the corresponding struts. Exemplarily $\lambda_{A}$ and $\lambda_{C}$ are visualized with the corresponding center points $G_{A,0}$, $G_{A,1}$, $G_{C,0}$, and $G_{C,1}$.

Although all parts of the Jig Maker were fabricated using precise CNC machining and the fits have tight tolerances, remaining manufacturing and assembly tolerances cause inaccuracies. Therefore, the actual positions of all relevant parts were measured after assembly of the device using the CMM. This measurement included the position of all six spheres of the base joints $G_{i,0}$ plus the axis of the alignment bolt to determine the true spatial relationship of the stationary part; the position of all platform joints $G_{i,1}$ and their spatial relation to the MCI on top of the mounting table for a measure of the movable part. These measures were used to build up a CAD assembly model of the Jig Maker (Fig. 4). Modeling of the movable platform was completed by an additional axis that represents the planned trajectory. This axis was defined by two working points, equivalent to the start and target point, whose coordinates are parameters that have been associatively linked to an Excel spreadsheet to drive the orientation of the trajectory axis within the CAD model of the Jig Maker based on the individually planned coordinates of the drilling trajectory. Assembly constrains were used to force the trajectory axis to be in alignment with the axis of the alignment pin.

Driven dimensions between corresponding centers of $G_{i,0}$ and $G_{i,1}$ provide the resulting strut lengths $\lambda_{i}$ for a specific trajectory (Fig. 3). A specific configuration of the Jig Maker can by determined by changing the coordinates of start and target point stored in the Excel spreadsheet and updating of the associated Inventor model. Equations (1) and (2) were used to calculate the corresponding values for rough and fine setting of the mechanical struts (with floor being a function that rounds down to the next integer).

$$\lambda_{i,\text{rough}}=\text{floor}(\frac{\lambda_{i}-239.00\;\mathrm{mm}}{10});$$ (1)

$$\lambda_{i,\text{fine}}=\lambda_{i}-239\;\mathrm{mm}-10*\lambda_{i,\text{rough}.}$$ (2)

### 2.2 General Concept and Workflow

The proposed concept of an individually moldable MSF requires the main activities listed below.

1. The universal reference frame is attached to the patient’s skull close to the location of the drilling. Therefore, three small skin incisions are made to enable direct bone-anchorage of the Trifix using three self-drilling and self-tapping bone screws.
2. Computed tomography imaging is obtained. The image has to cover both the target region and the whole reference frame.
3. Spherical reference markers are detected within the captured image. In addition, a straight path from the skull surface to the target point is planned.
4. Required strut lengths of the Jig Maker are calculated using the planned trajectory.
5. Length of each strut of the Jig Maker is adjusted.
6. All parts of the surgical template are aligned using the Jig Maker and permanently fixed using bone cement.
7. After hardening of the bone cement, the surgical template is removed from the Jig Maker and mounted on top of the reference frame.
8. The drill bit is equipped with a set collar for mechanical limitation of the drilling depth when the set collar reaches the top plane of the exterior drill bushing. The individual distance to the drill’s tip results from the target point of the planned trajectory.
9. The surgical procedure, here more precisely the drilling of the access to the middle ear cavity, can be performed.

The listed activities are of general validity when using the proposed MSF, regardless of the specific surgical application. It might be necessary to adapt or to extend the procedure in one or more details. For example, in CIS additional preparation of the bed for the internal receiver and insertion of the electrode array is required. As application-specific tasks like these come on top of the micro-stereotactic drilling procedure they have not been mentioned.

Please note that activity 8 was not performed in this study. Omitting of depth control was necessary as our method of determining the drilling accuracy at the level of the target point using the “ring-shaped target phantom” (RTP) requires a through-hole. In our opinion, accurate control of the drilling depth is less critical and less technologically challenging than providing sufficient radial accuracy with only small or negligible lateral deviation from the planned trajectory—which is especially true when passing the narrow region of the facial recess. Therefore, highly accurate determination of the lateral deviation by measuring the outlet of the drilling using the RTP was considered to be of higher importance than applying a depth control mechanism.

### 2.3 Experimental Evaluation

#### 2.3.1 Sample preparation and trajectory planning

Five half-skull phantoms (1339-28-1, Sawbones Europe AB, Malmö, Sweden) made of bone-substitute material (BSM) were used in this study; each of them to perform two separate drilling experiments. An artificial skin phantom (1485-185, Sawbones Europe AB) was added to achieve a more realistic simulation of the whole surgical workflow.

**Fig. 5:**
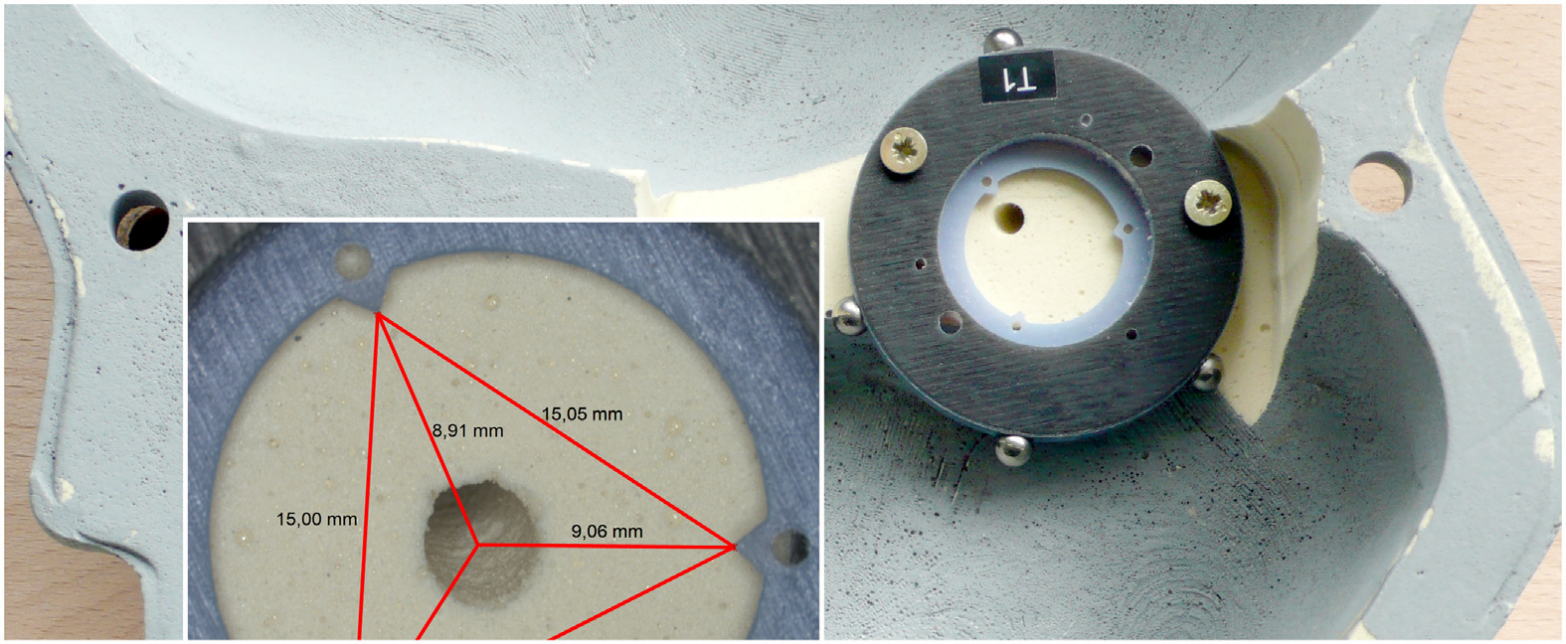
Ring-shaped target phantom. The RTP was screwed on the skull phantom after removing material in the region of the petrous apex to place the RTP at the natural position of the inner ear. Attached titanium spheres improve visibility in CT images and serve for registration purposes. The inward-looking tips of the RTP were used to determine the position of the bore hole by triangulation (see inset with exemplary measurements).

As the half-skull phantoms do not include anatomical structures, we attached an artificial target structure (Fig. 5), referred to as RTP in the following.^20^ The RTP consists of a plastic ring containing three triangular, inward-looking tips, which serves as reference landmarks for the evaluation of the drill hole position using microscopic imaging. Additionally, the RTP was equipped with four titanium spheres, which have been used to define the target coordinate system $\{\mathrm{CF}{\}}_{\mathrm{RTP}}$ as their position can be determined in both physical (using the CMM) and image space. Two screws fix the RTP on the inside of skull phantom.

Step 1 and 2 of the workflow in Sec. 2.2 are performed, by use of three bone screws (Fig. 1) and cone-beam computed tomography (CBCT, xCAT, Xoran Technologies LLC, Ann Arbor, Michigan) with an isotropic voxel size of 300  μm.

**Fig. 6:**
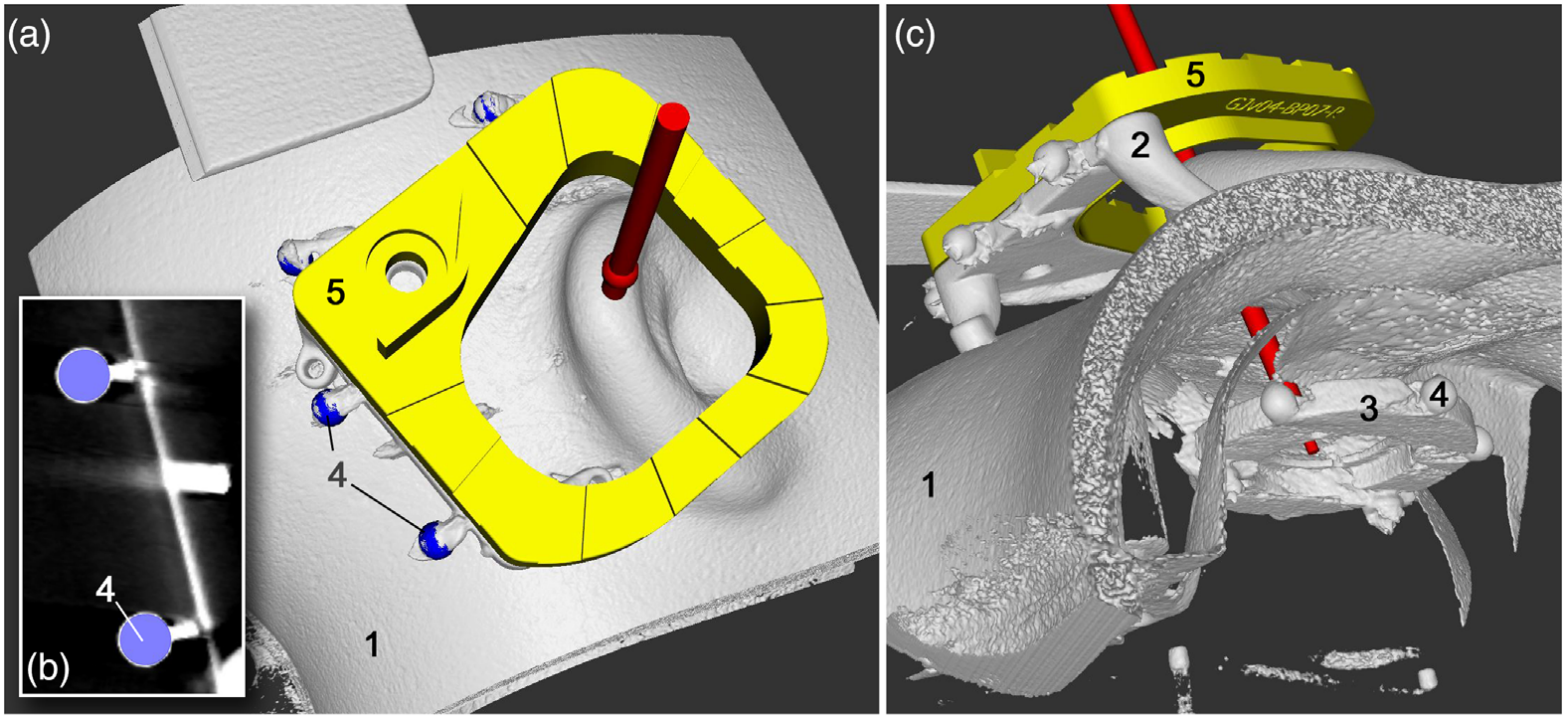
(a), (c) Two surface renderings and (b) one slice of CBCT imaging with additional rendered objects visualizes the half-skull phantom with silicone skin (1), the reference frame (2), the RTP (3), and spherical registration markers (4). The base plate (5) is visualized to show the available workspace during trajectory planning. Target point was planned at the level of the RTP (3).

A previously developed custom planning software^20^ was employed for image registration and trajectory planning. Semi-automated sphere detection was performed to localize the position of the fiducials of the Trifix. Their positions were registered to the CMM measured positions in physical space. For each trajectory, an entry point at the outer surface of the skull phantom and a target point at the inner surface somewhere between the tips of the RTP was planned (Fig. 6). Care was taken to prevent collision of both trajectories planned in the same skull phantom. Finally, the coordinates of the entry point $p_{e,\text{plan}}$ and target point $p_{t,\text{plan}}$ of the planned trajectory $t_{\text{plan}}$ were stored in the above mentioned Excel spreadsheet, which drives the associated Inventor assembly model of the Jig Maker. The planned trajectory was also used to calculate the intersection with the xy plane of ${\left\{\mathrm{CS}\right\}}_{3\text{fix}}$, which is referred to as starting point $p_{s,\text{plan}}$ in the following.

#### 2.3.2 Experimental evaluation with customization of the Jigs

**Fig. 7:**
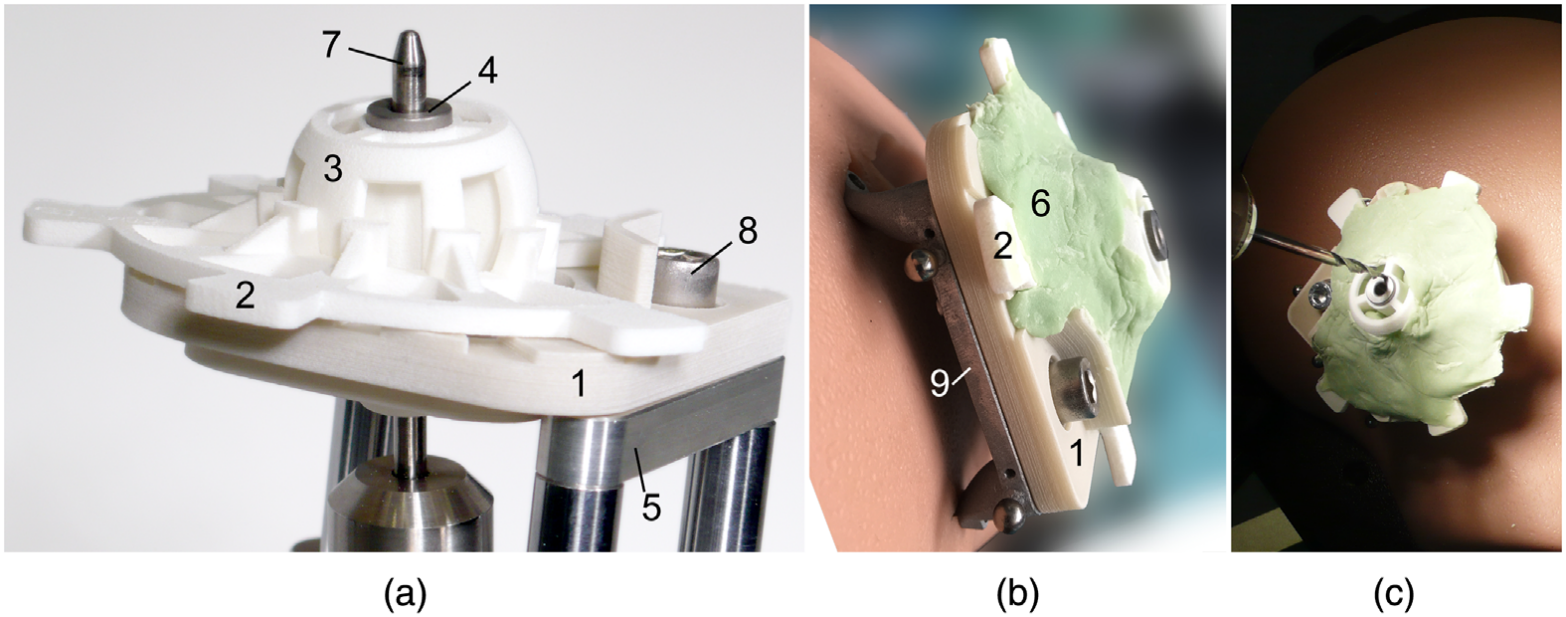
Moldable surgical targeting system. (a) Base plate (1), subcarrier (2), and drill bushing holder (3) with headed drill bushing (4) on top of the mounting table (5) of the Jig Maker before applying the bone cement (6). Alignment pin (7). Security screw (8). (b) Surgical template after customization on top of the skull-mounted reference frame (9, Trikfix). (c) The surgical targeting system before starting the drilling along the rigidly determined trajectory.

After updating the virtual model of the Jig Maker based on the planned trajectory, the corresponding lengths of all struts could be read out and were transferred into length setting values using (1) and (2). The length of each strut was adjusted manually, controlled by a second operator, and photographically documented. Subsequently, the base plate, the subcarrier, and the drill bushing holder were placed on top of the mounting table and temporarily aligned by the alignment pin (Fig. 7). Bone cement (Palacos MV 1×40, Heraeus Medical GmbH, Wehrheim, Germany) was prepared by mixing the two components manually as recommended by the manufacturer. At the beginning, the bone cement was applied using a spatula and was roughly distributed over the parts of the surgical template. With increasing viscosity, the bone cement could be spread and molded by use of the fingers. This was done to ensure infiltration of the provided cavities of the prefabricated parts.

After hardening of the bone cement the surgical template was mounted onto the Trifix [Fig. 7(b)], which was still attached to the half-skull phantom, to perform the drilling [Fig. 7(c)]. A custom-made twisted step drill bit (Kaestner-Tools GmbH, Steinbach-Hallenberg, Germany) with 4 mm in diameter at the basal part and 1.8 mm in diameter at the tip was chucked into an ordinary cordless drill/driver tool (GSR 10,8 V-LI-2, Robert Bosch GmbH, Stuttgart, Germany). Drilling was performed manually and slowly. Drilling depth was not controlled within the present study due to reasons mentioned above; instead, it was drilled until the drill bit fully exited the bone substitute to create a visible exit hole representing the drilling trajectory at the level of the RTP.

#### 2.3.3 Determination and analysis of errors

After each drilling experiment measurements were performed using the CMM to get insights of error components and their contribution to the total error. Measurements of the jig were collected immediately after the drilling experiments. It was assumed from a previous study^18^ that distortions appear over the course of time.

##### Length setting error

Lengths of all struts (in total 60 values; six struts in each of the 10 trials) were measured and compared with the desired lengths. Resulting deviations, herein referred to as “length setting error” ($\varepsilon_{\mathrm{LSE}}$), are a measure how accurately and reliably the hexapod can be operated manually, which means how good the user is able to adjust the length of the micrometer screws using the provided length setting values. The value $\varepsilon_{\mathrm{LSE}}$ also includes mechanical inaccuracies such as nonlinearities in the thread and fabrication tolerances of the struts including the magnetic ball joints, which could not have been fully eliminated by the calibration.

##### Pose setting error

Due to the kinematics of a Gough–Stewart platform, deviations of the strut’s length cannot be intuitively converted into pose errors of the moving platform. Therefore, the actual configuration of the Jig Maker was measured by determining the actual pose of the alignment pin $a_{\text{pin}}$ using the MCS defined by the mounting table. In contrast to the planned trajectory, the actual axis of the alignment pin represents the trajectory based on which the surgical template was actually fabricated; the deviation between both is hereafter referred to as setting error ($\varepsilon_{\text{set}}$). The deviations between $t_{\text{plan}}$ and $t_{\text{set}}=a_{\text{pin}}$ were measured using two planes perpendicular to $t_{\text{plan}}$ at the level of the start and target point, labeled $\Pi_{s}$ and $\Pi_{t}$, respectively. The intersecting of $t_{\text{set}}$, provided by the CMM measurement, with $\Pi_{s}$ and $\Pi_{t}$ leads to $p_{s,\text{set}}$ and $p_{t,\text{set}}$ with $\varepsilon_{t,\text{set}}={\left\|p_{t,\text{set}}-p_{t,\text{plan}}\right\|}_{2}$. To provide insights into the spatial distribution of the different error sources, the x and y axis of ${\left\{\mathrm{CS}\right\}}_{3\text{fix}}$ was projected into $\Pi_{s}$ and $\Pi_{t}$ to measure also the components of the errors in the corresponding directions.

##### Total positioning error

In the next step, the pose of the drill bushings within the surgical template was measured using the CMM as it represents the accuracy of the MSF regarding positioning of a surgical instrument at the desired target point. By pivoting the spherical probe of the CMM around the openings of the drill bushings, two points were determined describing the actual axis $t_{\mathrm{pos}}$ of the instrument guide. To provide the positions of the pivot points in ${\left\{\mathrm{CS}\right\}}_{3\text{fix}}$, the mounting table was used as holder for the surgical template. For the determination of the positioning error $\varepsilon_{\mathrm{pos}}$, the previously defined planes $\Pi_{s}$ and $\Pi_{t}$ were used again. In addition, the positioning inaccuracy caused by the jig itself was calculated by $\varepsilon_{t,jig}={\left\|p_{t,\mathrm{pos}}-p_{t,\text{set}}\right\|}_{2}$, therefore using the actual position of the alignment pin $t_{\text{set}}$ as new reference.

##### Total drilling error

The total error of the drilling experiments was measured using two different methods. First, the above-mentioned RTP was utilized. After drilling, an image of the drill hole inside the RTP was captured using reflected-light microscopy (Leica APO Z6, Leica Microsystems GmbH, Wetzlar, Germany) with full apochromatic optic (objective 0.5× Apo, Z6/Z16, f=187  mm) and a digital CCD camera (DFC 420, Leica, resolution: 2592×1944  pixels). The skull phantom was fixed in a special holder to ensure horizontal orientation of the RTP with respect to the microscope’s optical axis to avoid perspective distortions. Distances between the triangular tips and the center of the drill hole were measured and used to calculate the position of the target point $p_{t,\text{drill}}$ within ${\mathrm{CS}}_{\mathrm{RTP}}$ to determine $\varepsilon_{t,\text{drill}}^{\mathrm{RTP}}$. The calculation is described more detailed in Ref. 20. As this method only applies to the target point, it was not possible to determine the trajectory of the drilling $t_{\text{drill}}$. That is why there is no information about the size of the deviation at the start point.

Second, the CBCT was utilized to evaluate the drilled skull phantoms with the custom-made planning tool. Within the image data, the start and end point of the drilling were visually determined and used to define the trajectory of the drilling $t_{\text{drill}}$ after image registration using the reference frame. $\Pi_{s}$ and $\Pi_{t}$ were used again to calculate $p_{s,\text{drill}}$ and $p_{t,\text{drill}}$ and the Euclidean distances to $p_{s,\text{plan}}$ and $p_{t,\text{plan}}$ to provide a measure for the drilling error $\varepsilon_{\text{drill}}^{\text{CBCT}}$ at both locations.

## 3 Results

In total, 10 individual surgical targeting systems have been produced following the described workflow and successfully tested in drilling experiments. On average, 37.7±2.8  g of bone cement was used to build the customized surgical templates. No major complications with impact on accuracy and no unforeseen occurrences have been observed. The whole procedure was easily performable, took about 20 min per sample (including adjustment of the Jig Maker, template fabrication, and drilling), and does not require special skills of the users. Only manual length setting of the hexapod’s struts requires familiarity with the scales of the micrometer screws and therefore demands individual training. The current design of the reference frame limits access to the mastoid surface, which might conflict with preparation of the implant bed and placement of the internal receiver.

Length setting of the struts was possible with a mean accuracy of −0.09±0.09  mm, including two outliers with −0.65 and −0.35  mm.^19^ In these two cases (3.3%), length setting error was caused by mix-up of two digits when setting the micrometer screws. In all 60 cases, the struts were set a bit too short, indicating a systematic error in the calibrated virtual model of the Jig Maker.

The length setting errors caused a deviation $\varepsilon_{t,\text{set}}$ of the Jig Maker from the planning of on average 0.21±0.22  mm. The resulting mean positioning error $\varepsilon_{\mathrm{pos}}$ was found to be 0.27±0.14  mm at the level of the start point trajectory and increases to 0.41±0.30  mm at the target. The isolated positioning inaccuracy of the jig $\varepsilon_{t,jig}$ was 0.33±0.14  mm (assuming a faultlessly operating Jig Maker).

Finally, the accuracy of drilling along a previously planned trajectory was investigated. Utilizing the newly designed surgical template it was possible to reach the target point with a mean accuracy of 0.35±0.29  mm or 0.38±0.27  mm when using the second CBCT acquisition or the RTP, respectively, for evaluation.

All results are summarized in Table 1.

**Table 1:** Summary of results. The second column of the table indicates the method used to determine the corresponding error values. In the last colum, MW* and SD* are calculated without the two trials with incorrect manual length setting. In addition, the 95% confidence intervals for the most relevant mean values are provided in parenthesis.

	Method	#01	#02	#03	#04	#05	#06	#07	#08	#09	#10	MW ± SD	MW* ± SD*
$\varepsilon_{s,\text{set}}$	CMM	0.10	0.65	0.10	0.20	0.19	0.22	0.12	0.23	0.16	0.14	0.21±0.16	0.16±0.05
$\varepsilon_{t,\text{set}}$	CMM	0.24	0.80	0.06	0.17	0.08	0.19	0.07	0.20	0.07	0.19	0.21±0.22 (0.03, 0.38)	0.14±0.07 (0.08, 0.19)
$\varepsilon_{s,\mathrm{pos}}$	CMM	0.11	0.60	0.16	0.20	0.34	0.23	0.18	0.30	0.24	0.35	0.27±0.14	0.22±0.07
$\varepsilon_{t,\mathrm{pos}}$	CMM	0.44	1.05	0.39	0.44	0.18	0.17	0.29	0.06	0.30	0.78	0.41±0.30 (0.17, 0.65)	0.28±0.14 (0.18, 0.39)
$\varepsilon_{s,\text{drill}}$	CBCT	0.05	0.99	0.35	0.22	0.59	0.66	0.18	0.21	0.02	0.70	0.40±0.32	0.29±0.23
$\varepsilon_{t,\text{drill}}$	CBCT	0.40	1.08	0.41	0.21	0.30	0.21	0.18	0.18	0.05	0.46	0.35±0.29 (0.12, 0.58)	0.24±0.12 (0.15, 0.33)
$\varepsilon_{t,\text{drill}}$	RTP	0.11	1.10	0.28	0.19	0.23	0.31	0.47	0.34	0.42	0.40	0.38±0.27 (0.17, 0.60)	0.29±0.12 (0.21, 0.38)

## 4 Discussion

A minimally invasive approach to the inner ear requires high accuracy when drilling a single drill hole from the surface of the skull directly into the cochlea. It is known from previous studies^12^^,^^15^ that with rigidly attached MSF mandatory accuracy can be achieved. Furthermore, several concepts for image-guided, robot-assisted minCIS have been described^3^^,^^6^^–^^8^ of whose the robot system HEARO already underwent a first clinical trial and reached an accuracy of 0.21±0.09  mm at the level of the facial recess.^3^ However, most of these approaches require expensive equipment, specially trained technicians in the OR to run the devices,^3^^,^^6^^,^^12^^,^^15^^,^^21^^,^^22^ necessitate an additional previous surgical procedure for placement of bone anchors,^11^^,^^12^ or include a time-consuming sterilization step prior to Refs. 5 and 11, or during surgery.^4^ Motivated by these limitations, our research is focused on developing an easy-to-use solution for accurate instrument guidance with intraoperative template fabrication under sterile conditions.

In principle, there are two basic ways to enable surgical guidance. The first one comprises frameless methods [a.k.a. “image-guided surgery” (IGS)], where the instrument is tracked continuously and its spatial relation to the target object is visualized for the surgeon. In contrast, the second basic principle is frame-based. Here, a customized instrument guidance is utilized, which constrains the surgical instruments rigidly along a predefined trajectory targeting the anatomical region of interest. Well-known implementations of this concept are stereotactic frames widely used in neurosurgery and surgical templates for dental implant positioning. Although we have also explored frameless approaches incorporating IGS systems,^6^^,^^7^^,^^23^ we have opted for the frame-based approach in the past due to the prospect of greater accuracy based on experiences with both concepts.^6^^,^^24^

Basic condition is a stable, immobile spatial connection to bony structures. Stereotactic frames are hold in place by sharpened pins that indent the skull from opposite sides. Surgical templates for dental or maxillofacial purposes use the teeth or edentulous arches as protruding natural fixation regions. The STAMP method,^5^ originally developed for registration purposes, uses the individual surface topography of the temporal bone. An individual plate is planned and 3D-printed whose inner surface matches the patient’s individual bone surface structures. In the meantime, the concept was adapted to be used as a drill guide in the context of Bonebridge implantation (BB-STAMP^25^). However, as the size of the template is limited to the exposed bone and accuracy of this form-locking surface registration method decreases with reduced size of the exposure and number of unique surface features, it is obvious that the STAMP-method will provide only limited accuracy in minimally invasive approaches. Indeed, accuracy reported with the STAMP method is not sufficient for minCIS.^5^^,^^25^

Therefore, for MSFs that are localized to a small region of the skull and should provide highest accuracy one or more bone screws are required. To simplify the customization process, independent placement of three bone anchors was omitted, in contrast to previous described MSFs^4^^,^^11^^,^^12^^,^^15^ because this leads to a not standardized MCI. Adaption of the shape of the underside of the template to the varying location of multiple anchor points, in turn, is only possible when using rapid prototyping or CNC milling equipment. This leads to a remarkable delay due to the time-consuming fabrication process and following sterilization. Instead, bone anchorage was realized by a uniform supporting and fixation platform that provides a standard coupling interface (MCI) for template fixation. The function of this reference frame is similar to the prepositioning frame designed by Kratchman et al.,^10^ the universal frame by Vollmann et al.,^13^ the modified retractor used by Balachandran et al.,^26^ and the intermediate positioning frame described by Dillon et al.^27^

The main feature of the present concept is gluing disposable parts together. This significantly reduces the number and complexity of parts in comparison to joints that can be locked by mechanical fasteners, such as screw couplings, and clamp-connections. This is considered to be advantageous as fewer and simpler parts simplify both the requirements for the sterilization procedure and their intraoperative handling. In addition, tightening of fastening elements can introduce stresses in the clamped parts, which are sources of inaccuracy. Finally, gluing (in comparison to fastening) offers more flexibility regarding the configuration of the jig and the reshaping of the disposables parts when adapting to other surgical applications.

In its current version, the Trifix is made of aluminum for cost reasons. For surgical practice, the reference frame may be fashioned of other materials such as titanium, which provide higher rigidity and less artefacts in CT imaging. However, in this study, rigidity of the frame was not found to be a critical aspect. Rather, the stiffness of the base plate may affect the overall accuracy especially in the case of process forces during drilling. As it is only partly supported by the reference frame, higher forces may cause some bending within the surgical template, which could lead to deviations at the target point. This needs to be investigated in an additional study as the current study design does not allow for a separate analysis of this aspect. In the future, all separate parts (base plate, subcarrier, and drill bushing holder) will be designed to be disposables that will be delivered sterile to the operation room. However, for the present study the prototype was produced non-sterile out of polyamide using 3D printing technology due to cost reduction.

The conducted investigation of the length setting error showed that the usage of a manually operated Jig Maker is admissible. On average, adjustment of the strut’s length was possible with sufficient accuracy. The remaining deviation is indicating a systematic error that can be further reduced by an improved calibration of the strut’s length setting mechanisms in particular. However, two cases of misadjusted struts also show that there is an intrinsic risk for individual errors, which impacts patient’s safety when using a passive Jig Maker—although four-eye-principle was already implemented. Dominant error source was found to be the reading of the scales of the micrometer screws. One option is to replace manual length setting by an automated hexapod—accompanied by the drawback of higher costs regarding development and acquisition of the system by the hospitals. In the case of keeping the concept of a manually operated Jig Maker, an improved length setting mechanism needs to be implemented, but also additional security measures are desirable to detect fatal errors in manual length setting—at least before the surgical template is used.

Regarding the positioning accuracy, the achieved results are encouraging and support our previous findings (0.30±0.25  mm; n=18, reported in Ref. 18). With 0.41±0.30  mm, the average positioning error was slightly larger in this study. This difference might be explained by different measurement methods: In contrast to the earlier study, the accuracy was determined using a CMM and not a mechanical accuracy test bench. In addition, the sample size in the current study is smaller and the comparatively strong impact of two outliers in this study could be an additional explanation. Without the outliers the positioning accuracy ($\varepsilon_{t,\mathrm{pos}}$) is 0.28±0.14  mm, which is comparable to what we have found in Ref. 18. This value also represents a future scenario where misalignments can be detected by a safety measure and therefore the surgical template can be rebuilt until sufficient accuracy is proven. The 95% confidence interval for the mean positioning error at the target including all trials is (0.17 mm, 0.65 mm), whereas it decreases to (0.18 mm, 0.39 mm) when excluding the outlier cases.

When comparing $\varepsilon_{t,\text{drill}}^{\mathrm{CBCT}}$ with $\varepsilon_{t,\text{set}}$ (0.35±0.29  mm versus 0.21±0.22  mm), it becomes obvious that the inaccuracy of the alignment device has a substantial impact on the total drilling accuracy. As there are automated hexapods on the market, which operate with accuracy better than one micrometer, it is obvious that this new concept of a moldable surgical template offers room for further improvements and improved accuracy. If one assumed a faultlessly temporary pose setting procedure, the total drilling accuracy of the individualized jig could be reduced to 0.26±0.12  mm (${\varepsilon^{\prime }}_{t,jig}={\left\|p_{t,\text{drill}}-p_{t,\text{set}}\right\|}_{2}$) with the current design. The 95% confidence interval for the mean of this adjusted error value is (0.17 mm, 0.35 mm).

Furthermore, bone cement is suspected to cause shrinkage^18^ that impacts accuracy over time. In a further study, it is planned to reduce the amount of bone cement or even replace it by another adhesive to overcome bone cement related inaccuracies. As the bone cement is not a load-bearing component (in contrast to the device proposed by Ref. 13), the generous use of it, as performed in this study, is probably not necessary for producing a rigid surgical template. Kratchman and Fitzpatrick^12^ had already demonstrated that accuracy in the range of <0.2  mm is possible using an automated alignment device and low-viscosity, fast-curing cyanoacrylate adhesive. In contrast to our proposed design, the Freeze Frame is composed of much more separate parts. Each leg is adjusted independently one after the other using a CNC machine, which often might not be available close to the OR. These multiple steps of temporary pose setting for every leg are each pronounced to errors that could add up to a higher total pose setting error. In general, keeping the system as simple and the number of parts as low as possible seems to be beneficial.

The determined accuracy of the moldable surgical targeting system is comparable with other devices explored in the context of minimally invasive approaches at the lateral skull base. Table 2 summarizes the literature review. Note that the accuracy values were taken from the respective publications but were determined in different ways. For the Freeze Frame,^12^ for example, accuracy was evaluated in 3D space; while in this study, only lateral deviations from the planned trajectory are available. Therefore, comparability is given only to a limited extent and the absolute numerical values can only serve for a rough comparison of the systems. While the upper part of Table 2 covers studies in which the positioning error was investigated, the lower part provides an overview on published results for drilling accuracy, which includes the impact of process forces during drilling in real or artificial bone material.

**Table 2:** Accuracy of surgical assistance systems in minCIS. Positioning error is determined without impact of drilling forces whereas drilling error includes deviations from drilling in inhomogeneous bone or homogeneous BSM.

Technology^a^	Mean ± SD	Max	n	Material^b^	Reference
Positioning error	—	—	—	—	—
MSF	0.75±0.63	—	19	—	13
MSF	0.45±0.15	0.81	8	—	28
MSF, this paper	0.41±0.30	1.05	10	—	$\varepsilon_{t,\mathrm{pos}}$
MSF	0.37±0.18	0.61	5	—	14
MSF	0.30±0.25	1.15	18	—	18
Robot	0.24±0.11	0.37	4	—	29
MSF	0.14±0.13	0.24	10	—	12
Total drilling error	—	—	—	—	—
Robot + IGS	0.78±0.30	1.26	10	TBS	6
MSF, this paper	0.35±0.29	1.08	10	BMS	$\varepsilon_{t,\text{drill}}^{\mathrm{CBCT}}$
MSF	0.31±0.10	0.45	6	TBS	15
Robot + IGS	0.21±0.09	0.34	9	Patient	3
Robot + IGS	0.15±0.08	0.26	10	TBS	22

a MSF: micro-stereotactic frame; IGS: image-guided surgery system.

b TBS: temporal bone specimens; BSM: bone-substitute material.

When comparing both evaluation methods for determining the drilling accuracy (using second CBCT imaging or the RTP with optical measurement), no remarkable differences were found. Based on our data, it is not possible to decide which measurement method is more precise. However, as both independent methods gave the same results there is high confidence that the determined drilling accuracy is reliable and not impaired by inaccuracies of the measurement method.

The main focus of this study was on technical questions whether the proposed MSF provides necessary accuracy for minCIS. A final surgical protocol covering all steps of the procedure has not yet been developed and several details are subject of ongoing discussions. One open question is at what point of the surgery the implant bed will be prepared. If the reference frame is already in place at that point, it must not limit access to the mastoid surface. Based on our findings, slight modifications on the current design (e.g., longer legs of the Trifix or smaller planar top face) might be necessary for a clinical application and needs to be evaluated in a more realistic setting using cadaveric head specimens.

Another issue is the target point of the drilling. Our current restriction of stopping the drilling after reaching of the middle ear is in agreement with the other mentioned surgical systems such as the Microtable ^4^ or the HEARO robot.^3^ Both systems are currently used for drilling the access to the middle ear cavity while opening of the inner ear and electrode insertion is done using a tympanomeatal flap as an auxiliary access to the tympanic cavity. At the moment, it is an unsettled question whether direct opening of inner ear is possible in a save and atraumatic manner. Direct cochlea access was shown in cadaver studies^6^^,^^15^ but avoided in the above-mentioned, first clinical studies. One option could be to target the drilling trajectory toward the round window membrane and to open of the cochlea using a micro-needle also guided by the surgical template (or future laser^30^ or drilling tools^31^). In addition, use of an (automated) insertion tool^32^ attached to the MSF and therefore operating along the same trajectory is under investigation.

The drilling accuracy of the presented system is within the range of published results but not yet good enough to fulfill the accuracy requirements for an atraumatic opening of the inner ear.^2^ In addition, we expect additional deviations if drilling is performed in heterogeneous bone tissue instead in homogenous artificial bone material. However, there are many other surgical procedures that do not demand for such high accuracy: Keyhole neurosurgical procedures, for instance, can be successfully performed with an accuracy between 1 and 1.5 mm.^33^ Deep brain stimulation electrode implantation can be conducted with frame-based techniques having a deviation of 1.2±0.6  mm but is even possible with frameless techniques having only an accuracy of 2.5±1.4  mm.^34^ Recently, Minchev et al.^35^ reported brain tumor biopsies using a novel, minimally invasive, robot-guided biopsy technique where the median targeting accuracy of 1.5 mm is an improvement compared to the standard procedure with 1.7 mm.

## 5 Conclusion

We proposed a new concept for a surgical targeting system, which is designed for intra-operative customization of the surgical template. The utilization of bone cement was proven to fulfill the demands of an easy, quick, and prospectively intraoperatively doable customization. The measured drilling accuracy is not yet appropriate to fulfill the requirements of atraumatic opening of the inner ear, but the system is more than sufficiently accurate for many neurosurgical procedures. In its current implementation, it already outperforms conventional stereotactic frames and IGS systems in terms of targeting accuracy; however, further possibilities for improvement were pointed out. A limiting factor is the reliability of manual operation of the alignment device, which is source of individual errors. To provide sufficient patient safety, sufficient training of the surgical team and additional security measures are desirable to detect fatal errors in manual length setting before the surgical template is built. Furthermore, for a clinical application (semi-)automated trajectory planning software will be required to overcome the time-consuming manual planning procedure used for these experiments.
